# Sexually dimorphic brain volume interaction in college-aged binge drinkers

**DOI:** 10.1016/j.nicl.2015.12.004

**Published:** 2015-12-12

**Authors:** Timo L. Kvamme, Casper Schmidt, Daniela Strelchuk, Yee Chien Chang-Webb, Kwangyeol Baek, Valerie Voon

**Affiliations:** a Department of Psychiatry, University of Cambridge, Cambridge, United Kingdom; b Behavioural and Clinical Neurosciences Institute, University of Cambridge, Cambridge, United Kingdom; c Cognitive Neuroscience Research Unit, Department of Communication and Psychology, Aalborg University, Aalborg, Denmark; d Center of Functionally Integrative Neuroscience, MINDLab, Aarhus University, Aarhus C, Denmark; e NIHR Biomedical Research Council, University of Cambridge, United kingdom

**Keywords:** BD, binge drinking, MRI, magnetic resonance imaging, AUDIT, Alcohol Use Disorders Identification Test, AUDs, alcohol-use disorders, PFC, prefrontal cortex, IFG, inferior frontal gyrus, HV, healthy volunteer, NIAAA, National Institute of Alcoholism and Alcohol Abuse, BDI, Beck Depression Inventory, STAI, Spielberger Trait Anxiety Inventory, UPPS-P, UPPS-P Impulsive Behavior, SPM, Statistical Parametric Mapping, MNI, Montreal Neurological Institute, ICBM, International Consortium for Brain Mapping, WBIC, Wolfson Brain Imaging Center, GLM, general linear model, SVCs, small volume corrections, FWE, familywise error, AAL, Automatic Anatomical Labeling, Adolescence, Alcohol, Binge drinking, Gender, Magnetic resonance imaging, Voxel-based morphometry, Neurodevelopment, Striatum

## Abstract

**Background:**

Binge consumption of alcohol is a major societal problem associated with important cognitive, physiological and neurotoxic consequences. Converging evidence highlights the need to assess binge drinking (BD) and its effects on the developing brain while taking into account gender differences. Here, we compared the brain volumetric differences between genders in college-aged binge drinkers and healthy volunteers.

**Method:**

T1-weighted magnetic resonance imaging (MRI) images of 30 binge drinkers (18 males) and 46 matched healthy volunteers (23 males) were examined using voxel-based morphometry. The anatomical scans were covaried with Alcohol Use Disorders Identification Test (AUDIT) scores. Whole brain voxel-wise group comparisons were performed using a cluster extent threshold correction.

**Results:**

Several large clusters qualified with group-by-gender interactions were observed in prefrontal, striatal and medial temporal areas, whereby BD females had more volume than non-BD females, while males showed the inverse pattern of decreased volume in BD males and increased volume in non-BD males. AUDIT scores negatively correlated with volume in the right superior frontal cortex and precentral gyrus.

**Conclusions:**

These findings dovetail with previous studies reporting that a state effect of BD in college-aged drinkers and the severity of alcohol use are associated with volumetric alterations in the cortical and subcortical areas of the brain. Our study indicates that these widespread volumetric changes vary differentially by gender, suggesting either sexual dimorphic endophenotypic risk factors, or differential neurotoxic sensitivities for males and females.

## Highlights

• We examined brain volumetric differences between genders in college-aged binge drinkers and healthy volunteers.
• Gender significantly moderated brain volumetric differences in prefrontal, striatal and medial temporal areas.
• The severity of alcohol use in binge drinkers is associated with brain volumetric changes in the superior frontal cortex and precentral gyrus.

## 1 Introduction

Across the United Kingdom and the United States, binge drinking (BD) or episodic excessive rapid intake of alcohol is common in young adults (Fuller and Hawkins, 2014, Guttormsson et al., 2012, Johnston et al., 2014). BD is associated with serious health-related, societal and economic consequences (Miller et al., 2007) and has been linked to risky behavior, such as drink driving, sexually transmitted diseases and premature death (Anderson, 2007). In the adolescent and young adult age group, BD serves as a heightened risk factor for later alcohol-use disorders (AUDs) (Chassin et al., 2002, Crabbe et al., 2011, Feldstein Ewing et al., 2014).

Relatively few studies have addressed the neuroanatomical differences associated with binge drinking in the developing brain. Using structural magnetic resonance imaging (MRI), we have previously reported that college-aged binge drinkers have increased ventral striatal volume compared to controls (Howell et al., 2013). There are also studies showing that college-aged binge drinkers have increased mid-dorsolateral prefrontal gray matter volume (Doallo et al., 2014), while other studies suggested a thinning of the mid-anterior cingulate cortex in binge drinkers compared to light drinkers (Mashhoon et al., 2014), which has also been characterized by altered neurochemistry (Silveri et al., 2014).

A recent study on adolescents which applied machine learning techniques in order to establish predictors of current and future binge drinking (defined as a minimum of three lifetime binge drinking episodes leading to drunkenness by age 14) found that current binge drinkers had smaller ventromedial prefrontal cortex (PFC) and left inferior frontal gyrus (IFG) volumes (Whelan et al., 2014). In contrast, future binge drinking was predicted by reduced bilateral superior frontal gyrus and greater volume of the right middle frontal gyrus and premotor strip. A recent longitudinal study found that adolescents who initiated heavy alcohol use showed thinning of the right middle frontal gyrus in conjunction with decreased white matter (Luciana et al., 2013). Similarly, another longitudinal study on initiators of heavy alcohol use in adolescents showed reduced left ventral diencephalon, left inferior and middle temporal gyri, and left caudate and brain stem structures compared to continuous non-users (Squeglia et al., 2014).

Volumetric alterations have also been observed in adolescents with alcohol use disorders (AUDs) which is a more chronic and severe condition compared to BD. Volume reductions in temporal lobe structures and the ventral striatum have also been reported in adolescent AUD. For example, it was shown that dual marijuana users and AUD have decreased left hippocampal volume (Medina et al., 2007). Similarly, other studies reported decreased left (Nagel et al., 2005) and bilateral hippocampal volumes (De Bellis et al., 2000) in AUD. This is convergent with our findings in binge drinkers showing an inverse correlation with alcohol severity and hippocampal, amygdalae and ventral striatal volumes (Howell et al., 2013). Studies on adolescent AUD have also found decreased volumes in the prefrontal cortices compared to controls (De Bellis et al., 2005, Fein et al., 2013, Medina et al., 2008).

A gender interaction has been reported in both BD (Squeglia et al., 2012) and AUD (Fein et al., 2013, Medina et al., 2008). Thus, relative to controls, AUD males had smaller thalamus and putamen volumes while AUD-females demonstrated the opposite pattern (Fein et al., 2013). Female binge drinkers exhibited larger PFC volumes compared to controls whereas male binge drinkers showed the opposite (Squeglia et al., 2012).

A large body of evidence thus suggests that alcohol use disorder and its subtypes are marked by brain-volumetric alterations in multiple cortical and subcortical regions. Since neuromaturation carries on through adolescence and into early adulthood (Casey et al., 2005, Giedd et al., 1999, Lenroot and Giedd, 2006, Sowell et al., 2004, Tamnes et al., 2010), the effects of neurotoxic insults between different age groups may be dissociable. The consequences of alcohol intoxication may be further dispersed in older age groups as alcohol consumption increases during this transition (Hermens et al., 2013, SAMHSA, 2014). We have previously published on volume differences in binge drinkers with a 3 month duration of BD (Howell et al., 2013). Here we intend to replicate these findings using a more stringent definition of BD with a 6 month duration and focus on the effects of gender. We hypothesized structural changes in ventral striatal volume as a function of binge drinking status and a gender-specific interaction.

## 2 Methods

### 2.1 Participants

Thirty binge drinkers and 46 healthy volunteers, matched for age and gender participated in the study (Table 1). Binge-drinking subjects were recruited through local advertisements in both community- and university-based areas in the East Anglia region while the healthy volunteers (HV) were recruited from the Behavioural and Clinical Neuroscience Institute healthy volunteer list and through local advertisements. The criteria used for BD were based on the National Institute on Alcoholism and Alcohol Abuse diagnostic criteria (NIAAA, 2004): consumption of ≥ 5 drinks and ≥ 4 drinks in a 2-hour period (for males and females, respectively) at least once a week for the last six months. Participants had to report getting drunk at least once a week during these binge drinking episodes. Participants were included if they were over 18 years old, had no history of regular or current use of other substances, and were free from any major psychiatric disorders (assessed with the Mini International Neuropsychiatric Inventory, Lecrubier et al., 1997). Those who were not suitable for MRI or presented major neurological illness, or head injury were not included in the study. All participants were asked to refrain from alcohol consumption at least 24 h before the scan and underwent a urine drug screen and an alcohol breathalyzer test. Written informed consent was obtained and the study was approved by the University of Cambridge Research Ethics Committee. Participants were reimbursed for their participation and travel expenses.

### 2.2 Data acquisition

Participants completed the Alcohol Use Disorders Identification Test (AUDIT) (Saunders et al., 1993), Beck Depression Inventory (BDI) (Beck et al., 1988), the State Trait Anxiety Inventory (Spielberger et al., 1983) and the UPPS-P impulsivity scale (Whiteside and Lynam, 2001). The BDI score of one healthy volunteer was > 30 and was removed from analysis.

Anatomical data were obtained using a Siemens 3T Tim Trio scanner (Siemens Medical System Systems, Erlangen, Germany) with a 32-channel head coil at the Wolfson Brain Imaging Center (WBIC) at the University of Cambridge. Magnetization prepared rapid gradient-echo sequence parameters were tailored to collect T1-weighted images with: repetition time = 2300 ms; echo time = 2.98 ms; matrix 240 × 256 × 176 mm and voxel size 1 × 1 × 1 mm.

### 2.3 Data processing

The 3D T1-weighted images were preprocessed with Statistical Parametric Mapping software (SPM8) (http://www.fil.ion.ucl.ac.uk/spm). The images were reoriented, aligning the origin approximately to the anterior commissure. New Segment was used to classify different tissue types (cerebrospinal fluid, white matter, and gray matter) using a tissue probability map to assign voxels a probability of belonging to one of these groups. The total intracranial volume for each participant was estimated on the basis of the summed volume of these tissue classes. DARTEL, a diffeomorphic method, was used to generate a sample-specific average template for the iterative alignment for non-linear deformation of each subjects' gray and white matter images (Ashburner, 2007). This template was then registered to the tissue probability maps using an affine transformation in the warping procedure. The images were registered in the ICBM 152 MNI space. All images were smoothed using a 10 mm full width at half maximum isotropic Gaussian kernel in the final normalization procedure.

### 2.4 Statistical analysis

The general linear model (GLM) was corrected for total intracranial volumes using proportional scaling. An explicit mask created from a binarized image from the SPM brainmask template using ImCalc was used.

Imaging data were analyzed with a 2 × 2 design with a factor of gender (female, male) and subject group variable (BD, HV) partialling out age. We also repeated the analysis with BDI as a covariate of no interest to ensure the findings were unrelated to depressive symptoms. We further examined the correlation between AUDIT and the dependent variable of brain volume for both BD and HV groups combined and separately within each group controlling for age and gender to assess the influence of alcohol severity.

We applied whole brain cluster extent threshold to assess significance. To determine the cluster extent threshold for the models aimed at investigating a gender interaction, we first viewed the data at whole brain uncorrected P < 0.001, however, as one cluster size was very large (cluster size = 47,963 voxels), we elected to view the data at a more stringent threshold; whole brain voxel-level uncorrected P < 0.0001 (Woo et al., 2014). Whole brain cluster-corrected familywise error (FWE) P < 0.05 was considered significant. Given our a priori hypothesis, we included a comparison using small volume corrections (SVCs) specifically for the ventral striatum. The ventral striatum region of interest template was hand drawn using MRIcro using the delineations of Martinez and colleagues (Martinez et al., 2001) and has been used in previous articles (Howell et al., 2013, Murray et al., 2008). For the ventral striatum, SVC FWE P < 0.05 was considered significant. For the AUDIT covariate analysis, whole brain cluster extent threshold FWE corrected P < 0.05 was considered significant.

Subjects' characteristics and questionnaire scores were compared using independent t-tests. Plots depicting the interaction were created by comparing the peak parameter estimate values of the significant regions using an ANOVA and utilizing Tukey's HSD tests to indicate significant differences. All statistical analyses on demographic, correlation data and plots were performed using R version (3.2.0) (R Core Team, 2014) with packages ggplot2 (v1.0.1).

## 3 Results

### 3.1 Participants

Thirty binge drinkers and 46 healthy volunteers participated in the study. There were no significant differences in participant characteristics, depression, and anxiety scores, except for a significant difference in impulsivity scores (Table 1). As expected, BD subjects had higher AUDIT scores compared to healthy volunteers (t(26,43) = 27.90, P < 0.001). There was no difference between BD males (mean = 16.25, SD = 5.31) and BD females (mean = 17.11, SD = 4.59) with respect to the severity of alcohol use (P = 0.67).

### 3.2 Imaging data

**Fig. 1:**
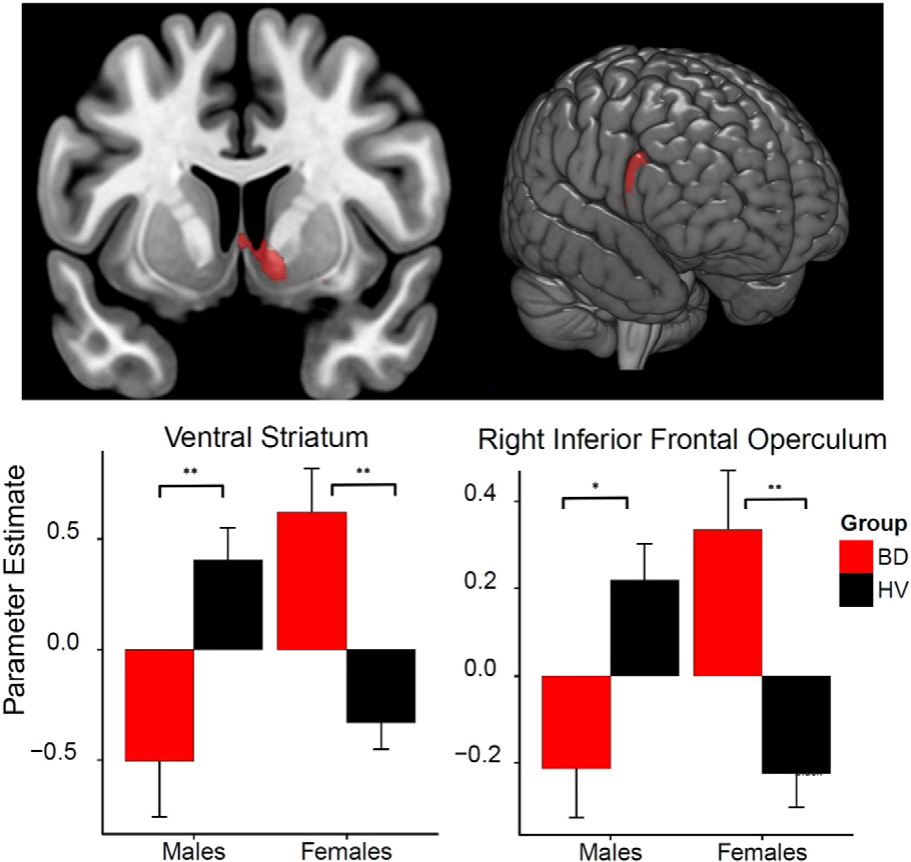
Volumetric group × gender interaction in the ventral striatum and right inferior frontal operculum. Brain images shown at P < 0.0001 uncorrected in the group by gender interaction: Left pane: peak parameter estimate for the ventral striatum MNI (x, y, z; mm):[12, − 1.5, − 10.5]; right pane: peak parameter estimate for right inferior frontal operculum MNI (x, y, z; mm):[60, 24, 27]; for binge drinkers (BD) and healthy volunteers (HV). *P < .01, **P < .001.

In the critical examination of a group × gender interaction, the factorial model revealed significance at cluster level in the following brain regions: prefrontal (bilateral inferior frontal cortex; right medial superior frontal cortex), striatal (left caudate and putamen), right fusiform gyrus (extending into bilateral hippocampus and ventral striatum), motor preparatory regions (right supplementary motor area) somatosensory cortex (right postcentral gyrus) and left middle temporal (Table 2). BD males had smaller volumes in these regions compared to HV males, and BD females had greater volume compared to HV females (Table 2). The group × gender interaction was also apparent using an SVC analysis focusing on the ventral striatum (MNI (x, y, z; mm) peak coordinates: 12.0, − 1.50, − 10.5, Z = 4.67 FWE-corrected P = 0.002; cluster size = 285) (Fig. 1). We controlled separately for BDI and AUDIT scores and showed that the group findings remained significant except for the clusters in the right frontal inferior operculum and left caudate. There were no significant regions when testing for the opposite interaction pattern. There was neither a main effect of group.

**Fig. 2:**
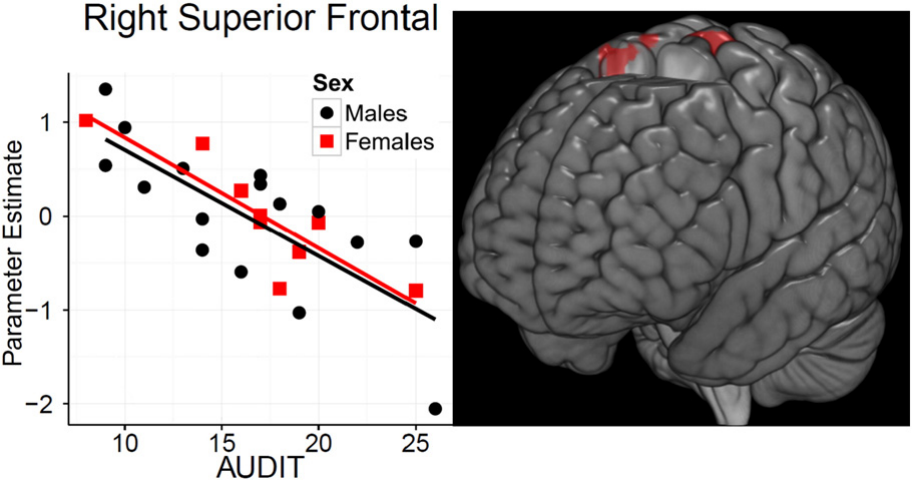
Right superior frontal correlated negatively with Alcohol Use Disorders Identification Test (AUDIT) scores. Left graph shows the negative correlation between the right superior frontal parameter estimate and AUDIT scores for males and females in the binge drinking (BD) group. Right: brain image shown at P < 0.0001 uncorrected.

There was no relationship between AUDIT scores and gray matter volume, whole brain FWE at P < 0.05. However, in the BD group alone, the right superior frontal [Z = 4.43, P = 0.009] and the left paracentral lobule [Z = 4.42, P = 0.014] showed a significant negative correlation with AUDIT as depicted in Table 3 and Fig. 2.

## 4 Discussion

### 4.1 Main findings

In this study, we show using voxel-based morphometry, a gender by disorder interaction modifying brain volume in college-aged BD relative to healthy volunteers. We show an interaction effect in prefrontal regions implicated in inhibitory control, motor preparatory regions, the left caudate and hippocampal and ventral striatum. Prior research within AUD has commonly demonstrated reductions in the medial temporal lobe and striatal structures in adolescents (De Bellis et al., 2000, Medina et al., 2007, Nagel et al., 2005, Squeglia et al., 2014), at the transition between adolescence and adulthood (Ozsoy et al., 2013) and in adulthood (Agartz et al., 1999, Beresford et al., 2006, Makris et al., 2008, Sullivan et al., 1995, Sullivan et al., 2005, Wrase et al., 2008). In the present study, we report findings in similar neuroanatomical structures with a binge drinking sample representing the college-age transitional period. Unlike the above mentioned studies addressing AUD the present study found no evidence for significant brain volumetric differences between healthy volunteers and binge drinkers separately within males and females. Instead, our main finding of a gender by disorder interaction is in line with similar studies on BD and AUD (Fein et al., 2013, Medina et al., 2008, Squeglia et al., 2012), showing a gender interaction in the thalamus, putamen and prefrontal cortex. Our findings extend this interaction to other neural regions relevant to alcohol misuse including inhibitory control, goal-directed behaviors, memory, reward and motivation processes. The direction of interaction was such that female binge drinkers had increased volume compared to healthy volunteers while male binge drinkers showed decreased volume. Most significant regions were present after controlling for differences in depression scores (BDI), alcohol severity (AUDIT), and age. This suggests that the BD status is associated with robust volumetric differences in the aforementioned regions irrespective of the severity of alcohol use and depressive symptomology. Moreover, in an examination of AUDIT as a covariate of interest we show that within binge drinkers, alcohol severity is inversely correlated with right superior frontal and left paracentral lobule volume. The finding that AUDIT scores in BD for both genders show a negative correlation in these regions and not the regions in the gender-by-group interaction suggests that state effects and dose–response relationships have dissociable associated neuroanatomy.

### 4.2 Gender interaction

The interaction finding is important as the specific pattern of gender interaction dovetails with recent papers examining a younger age group (m = 14.9 and m = 18.1 years old, respectively) (Fein et al., 2013, Squeglia et al., 2012). In contrast, in a younger population (15–17 years old) with more severe AUD, Medina and colleagues found the opposite interaction pattern, whereby AUD males had thicker prefrontal cortices compared with non-AUD males and AUD females had smaller volumes compared with non-AUD female controls (Medina et al., 2008). One possible explanation of the gender differences may be related to the disparate neuromaturation trajectories in the endocrine (Emanuele et al., 2001, Kim et al., 2003) and neuromodulatory systems (Guerri and Pascual, 2010, Vries, 1990) between genders, along with differences in alcohol metabolism and distribution of body fat (Wechsler et al., 1995) in males and females. Indeed, the demonstrated reversal of the gender effects between different age groups could be due to underlying gender-specific neuromaturational processes in the rate and timing of synaptic pruning (Giedd, 2004, Lenroot and Giedd, 2006), coupled with differences in neurotoxic sensitivity between genders. The present finding that female binge drinkers have increased volume compared to male binge drinkers, reverses the typical sexual dimorphic pattern present in healthy individuals (Lenroot et al., 2007), and fits with the hypothesis posed by (Squeglia et al., 2012) of a more pronounced impairment of healthy pruning in females due to alcohol exposure. In contrast, alcohol exposure may aberrantly enhance pruning in males. Prospective longitudinal studies into the anatomical differences of alcohol use subtypes compared to healthy volunteers are needed to further substantiate this hypothesis. Moreover, future studies should also focus on group × gender interactions.

If the morphological changes precede the binge drinking behavior, then it could serve as an endophenotypic risk profile available for the development targeted interventions (Conrod et al., 2008, Thush et al., 2007). As an example, it could be possible that the neuroanatomical substrates pertaining to psychological functions cause or facilitate the behavior and thereby warn about potential risk factors in a diatheses-stress conceptualization of mental health. In this regard, the observed gender interaction is a crucial additional qualifier to a endophenotypic risk factor of binge drinking. Further open questions include which aspects of addiction the structural changes are associated with such as the craving component, the intoxication, the lack of negative reinforcement during withdrawal or the maintenance of the binge drinking behavior (Koob and Volkow, 2010).

Alternatively, there may be psycho-social factors co-occurring with the specific gender by drinking group contributing to the volumetric interaction. While it remains entirely speculative, personality differences pre-existent to binge drinking could be open to a range of neuro-psycho-social interactions with gender that might result in the present neuroanatomical findings. Studies addressing gender difference in the factors that influence problematic drinking habits show that while some factors appear to affect the genders equally, other factors such as physiological response to alcohol, estimates of perceived peer alcohol use and the social sanctions of alcohol consumption differ between the sexes (Alfonso-Loeches et al., 2013, Petit et al., 2013).

### 4.3 Ventral striatal findings

An important aim of the current study was to replicate the findings of our previous study (Howell et al., 2013) showing increased ventral striatal volume in binge drinkers (controlled for gender) with a 3 month history of binge drinking. Our results suggest a partial replication, as we found no significant difference between groups, but rather a gender interaction such that only females had increased ventral striatal volume while males had decreased volume. There are several reasons why our findings may appear discrepant. First, our prior study had an unequal gender balance in which females outnumbered males nearly twofold, thus emphasizing an increase in ventral striatal volume whereas the current study is more equally balanced. Secondly, our prior study addressed the changes occurring in a population with limited exposure to binge drinking whereas the current study focuses on a more severe group with a 6 month history of binge drinking. When considering gender differences, the findings from both our studies confirm the hypothesis of greater ventral striatum volume in BD females. A recent study examining structural differences between adolescents with and without familial alcoholism also found increases in the left ventral striatum in females only (Cservenka et al., 2015).

### 4.4 Volumetric changes in inferior frontal gyrus, hippocampus and amygdala

Additionally, in the analysis of interaction, we found significant clusters in bilateral inferior frontal gyrus (IFG), regions implicated in both preclinical and clinical research in response inhibition (Aron et al., 2014, Swann et al., 2012). We note that a recent study in 14 year old adolescents showed that binge drinking was associated with lower left inferior frontal cortical volumes. Here our study emphasizes a divergent effect dependent on gender.

Our findings on the volumetric changes in medial temporal lobe are in line with multiple studies showing an association between structural alterations in the hippocampus and amygdala and alcohol consumption. In the adult AUD literature, a study examining the contrast between adolescent onset and late-onset alcohol use transitioning into adult alcoholism found reductions in right hippocampal volumes for the adolescent versus late-onset groups (Ozsoy et al., 2013). An examination of the alterations of the reward network structures carried out by (Makris et al., 2008) found decreased volumes in amygdala and nucleus accumbens in a sample of abstinent long-term chronic alcoholic men. Further studies have highlighted the reduction of gray matter volume in adult AUD, replicating findings in similar medial temporal lobe and striatal structures such as the amygdala (Makris et al., 2008, Wrase et al., 2008), hippocampus (Agartz et al., 1999, Beresford et al., 2006, Sullivan et al., 1995, Wrase et al., 2008) and ventral striatum (Sullivan et al., 2005, Wrase et al., 2008).

### 4.5 Evidence from longitudinal studies

Given the cross-sectional nature of the present study, hypotheses about cause and effect must remain entirely speculative, as the observed pattern of volumetric alteration may precede the BD behavior or they may be a function of the neurotoxic consequences of alcohol insults. Even so, the findings of a significant interaction in bilateral IFG is compelling as (Whelan et al., 2014) found reduced volume in the left IFG to be a robust classifier in discriminating current binge drinkers from non-binge drinkers. Whelan and colleagues constructed logistic regression models from a large sample (n = 692) in order to identify predictors able to discriminate future binge drinkers from controls by age 14. As such, their work represents the most comprehensive account of endophenotypic biomarkers for the development of BD to date. Moreover, the present reported finding of an inverse relationship between AUDIT and volume in the right superior frontal gyrus in binge drinkers fits with the finding of reduced superior frontal gyrus as a predictor of future binge drinkers (Whelan et al., 2014). Similarly, a recent article found binge drinkers to have reduced cortical thickness in this region (Mashhoon et al., 2014). Diffusion tensor imaging studies have also found disruptions of white matter integrity in the left superior frontal in adult alcoholic patients (Konrad et al., 2012) and in adolescents initiating alcohol compared to non-initiators in a two year longitudinal study (Luciana et al., 2013). We find a relationship between alcohol severity and right superior frontal gyrus volume within BD, convergent with reports of a state effect associated with current and predicted BD status.

### 4.6 Strengths and limitations

The principal strength of the current study is its use of more stringent criteria of BD status with a more pronounced 6 month duration of BD while focusing on a sparsely explored age-group. Further strengths of the study include the highly conservative calculation of voxel-wise significance threshold at P < 0.0001 (Woo et al., 2014) and the relatively large sample size compared to the aforementioned studies.

This study also displays several limitations that should be taken into account. While our primary aim was to investigate the interaction of gender on volumetric differences the current study failed to match the gender of binge drinkers completely, such that there were only 12 females and 18 males. Given our highly significant results we set the arbitrary voxel-level primary threshold at a more stringent P < 0.0001 for suprathreshold voxels, as is recommended for highly powered studies (Woo et al., 2014). Despite its purpose, which is to lower the probability of type I errors, the large size and amount of clusters reported does pose a disadvantage in terms of spatial specificity (Friston et al., 1994, Nichols, 2012, Woo et al., 2014). The shortcomings of topographical precision suggest that the exact loci of significant clusters in this study should be interpreted with caution. Furthermore, this study cannot make inferences about causality, which would be optimally addressed in longitudinal studies.

### 4.7 Conclusion

In conclusion, the current study sheds light on a more severe group of binge drinkers in a rarely studied age-group. In keeping with the literature on alcohol research into brain morphometry, we show that BD is associated with volumetric alterations in cortical and subcortical regions. Our data further indicates that this brain volume varies significantly as a function of gender, whereby BD males are marked by reductions in volume and BD females by increases. The effects may be related to differences in neuromaturation trajectories and neurotoxic sensitivities between males and females or due to endophenotypic risk factors for either gender. Further studies exploring endophenotypic risk factors and neuroarchitectural predictors of future binge drinkers, including adequate control such as unaffected family members or longitudinal studies are required in order to provide a comprehensive scientific explanation of these brain–behavior relationships.

## Author conflict of interest

The authors have no conflict of interests to declare.

## Author contribution

VV was responsible for the study design and experimental setup. DS, YCCW, and KB performed the experiments. Timo L. Kvamme and CS performed the analysis of the data. Timo L. Kvamme and VV interpreted the data, Timo L. Kvamme wrote the paper, Timo L. Kvamme and VV Revised the manuscript. All authors approved the final manuscript.

## Figures and Tables

**Table 1:** Demographic and behavioral data for healthy volunteers and binge drinkers.

	Binge drinkers (n = 30)			Healthy volunteers (n = 46)		
Females (n = 12) Mean (SD)	Males (n = 18) Mean (SD)	Total mean (SD)	Females (n = 23) Mean (SD)	Males (n = 23) Mean (SD)	Total mean (SD)
Age^d^	21.08 (1.78)	21.38 (2.83)	21.27 (2.43)	20.26 (1.28)	22.30 (2.05)	21.28 (1.98)
AUDIT^a^, ^b^, ^c^	17.11 (4.59)^f^	16.25 (5.31)^g^	16.56 (4.98)	4.04 (2.57)	5.10 (4.27)^g^	4.55 (3.48)
BDI	11.44 (8.92)^f^	9.50 (6.68)^g^	10.20 (8.01)	7.70 (6.36)	5.48 (6.05)^g^	6.63 (6.24)
STAI	41.11 (9.61)^f^	39.93 (10.80)^g^	40.36 (10.20)	39.86 (10.71)	41.11 (11.0)^e^	40.41 (10.72)
UPPS-P^a^, ^b^, ^c^	147.67 (20.60)^f^	140.06 (16.94)^g^	142.8 (18.29)	130.09 (15.60)	126.28 (20.70)^e^	128.41 (17.88)

AUDIT: Alcohol Use Disorders Identification Test, BDI: Beck Depression Inventory, STAI: Spielberger Trait Anxiety Inventory, UPPS-P: UPPS-P Impulsive Behavior, standard deviations in brackets.

All t-tests are for 2-tailed independent samples Welch's t-tests.

a Female binge drinkers ≠ female controls, P < .05.

b Male binge drinkers ≠ male controls, P < .05.

c Binge drinkers ≠ controls, P < .05.

d Female ≠ male, P < .05.

e Missing values from 5 participants.

f Missing values from 3 participants.

g Missing values from 2 participants.

**Table 2:** Significant volume differences in the group by gender interaction: (BD males ↓/HV males ↑/BD females ↑/HV females ↓).

Structure	Peak voxel MNI coordinates (*x*, *y*, *z*)	Z-score	Cluster extent	P-value FWE corrected	Extends into
R fusiform gyrus	(34, 2, − 36)	5.09	10,999	0.000	Ventral striatum L amygdala R temporal inferior L fusiform L & R hippocampus R parahippocampal
R supplementary motor area	(2, 6, 70)	4.66	1101	0.000	R frontal SUPERIOR
L temporal middle lobe	(− 54, − 18, − 20)	5.95	3380	0.000	L temporal inferior R fusiform
R frontal inferior operculum	(60, 24, 27)	4.69	698	0.002	L postcentral
L frontal inferior operculum	(− 62,14,16)	4.98	570	0.005	L frontal inferior triangularis
R postcentral gyrus	(24, − 39, 45)	5.49	2032	0.000	L precuneus
L precuneus	(− 16, − 51, 51)	5.06	1041	0.000	L precuneus R SMA R paracentral lobule
R Medial Superior Frontal Cortex	(8, 33, 64)	4.60	436	0.011	
L Caudate	(− 10, 24, 4)	4.37	535	0.006	L putamen

MNI: Montreal Neurological Institute, L: left, R: right. Results reported in the following tables using cluster extent thresholds were generated from statistical parametric maps at a threshold of P < 0.0001 whole brain uncorrected. Anatomical localizations were performed using the Automatic Anatomical Labeling (AAL) atlas (see Methods for more details).

**Table 3:** Volumetric correlations with alcohol severity.

Structure	MNI coordinates (*x*, *y*, *z*)	Z-score	Cluster extent	(FWE-corr) P-value	Extends into
R superior frontal	(15, 9, 73)	4.43	833	0.009	R SMA
L paracentral lobule	(− 15, − 16, 76)	4.42	756	0.014	L precentral

The table shows negative volumetric correlations between binge drinkers and alcohol severity (AUDIT: Alcohol Use Disorders Identification Test). MNI: Montreal Neurological Institute, L: Left, R: Right. Results reported in the following tables using cluster extent thresholds were generated from statistical parametric maps at a threshold of P < 0.0001 whole brain uncorrected. Anatomical localizations were performed using the Automatic Anatomical Labeling (AAL) atlas (see Methods for more details).
